# Brain metastasis in a patient with *BRCA2*-mutated treatment-related neuroendocrine prostate carcinoma and long-term response to radiotherapy and Olaparib: A case report and literature review

**DOI:** 10.1097/MD.0000000000037371

**Published:** 2024-03-01

**Authors:** Rio Uehara, Daisuke Obinata, Sho Hashimoto, Ken Nakahara, Hideaki Uchida, Tsuyoshi Yoshizawa, Junichi Mochida, Kenya Yamaguchi, Masakuni Sakaguchi, Yoshinari Ozawa, Fumi Mori, Katsuhiro Miura, Toshiyuki Ishige, Shinobu Masuda, Tomohiro Nakayama, Satoru Takahashi

**Affiliations:** a Department of Urology, Nihon University School of Medicine, Itabashi-ku, Tokyo, Japan; b Department of Radiology, Nihon University School of Medicine, Itabashi-ku, Tokyo, Japan; c Department of Neurological Surgery, Nihon University School of Medicine, Itabashi-ku, Tokyo, Japan; d Division of Hematology and Oncology, Department of Internal Medicine, Nihon University School of Medicine, Itabashi-ku, Tokyo, Japan; e Division of Oncologic Pathology, Department of Pathology and Microbiology, Nihon University School of Medicine, Itabashi-ku, Tokyo, Japan; f Division of Laboratory Medicine, Department of Pathology and Microbiology, Nihon University School of Medicine, Itabashi-ku, Tokyo, Japan.

**Keywords:** *BRCA2* mutation, castration-resistant prostate cancer, PARP inhibitors, treatment-related neuroendocrine prostate carcinoma

## Abstract

**Background:**

A new subtype of prostate cancer called treatment-related neuroendocrine prostate carcinoma (t-NEPC) was added to the revised World Health Organization classification of prostate cancer in 2022. t-NEPC cases are increasing, and there is no established standard treatment.

**Methods:**

A 49-year-old male patient was referred to our department for dysuria. A rectal examination and a prostate biopsy revealed stony hardness and prostate adenocarcinoma, respectively. Imaging studies confirmed the presence of multiple bone and lymph node metastases. The patient was started on upfront treatment with androgen deprivation therapy and an androgen receptor signaling inhibitor, which resulted in a significant (>90%) decrease in prostate-specific antigen (PSA) levels. The patient experienced postrenal failure 6 months later, attributable to local disease progression. Concurrently, there was an elevation in neuron-specific enolase (NSE) levels and an enlargement of pelvic lymph node metastases, without PSA progression.

**Results:**

Biopsy specimen for cancer genome profiling revealed deletion of *BRCA 2* and *PTEN*, AR amplification, and the presence of the *TMPRSS2-ERG* fusion gene. Based on increased NSE and *BRCA2* mutations, a diagnosis of t-NEPC with *BRCA2* mutation was eventually made. The patient received docetaxel chemotherapy and pelvic radiotherapy. Subsequently, he was treated with olaparib. His NSE levels decreased, and he achieved a complete response (CR). However, 18 months following the olaparib administration, brain metastases appeared despite the absence of pelvic tumor relapse, and the patient’s PSA levels remained low. Consequently, the patient underwent resection of the brain metastases using gamma knife and whole-brain radiotherapy but died approximately 3 months later.

**Conclusion subsections:**

Platinum-based chemotherapy is often administered for the treatment of t-NEPC, but there are few reports on the effectiveness of olaparib in patients with *BRCA2* mutations. In a literature review, this case demonstrated the longest duration of effectiveness with olaparib alone without platinum-based chemotherapy. Additionally, the occurrence of relatively rare, fatal brain metastases in prostate cancer after a long period of CR suggests the necessity of regular brain imaging examinations.

## 1. Introduction

Despite recent progress in treating castration-resistant prostate cancer (CRPC), some patients still have poor prognoses. One reason for changing cancer cells is their phenotypes, including transitioning to neuroendocrine prostate cancer (NEPC). Recently, a new subtype of prostate cancer called treatment-related NEPC (t-NEPC) was added to the revised World Health Organization classification of prostate cancer.^[1]^ On the other hand, CRPC with *BRCA* mutations has been the focus of much research due to its unique genetic makeup and challenging prognosis.^[2]^ A recent study showed that olaparib, a polyadenosine diphosphate-ribose polymerase (PARP) inhibitor, is a promising treatment option for CRPC with *BRCA* mutations, surpassing the efficacy of androgen receptor-signaling inhibitors.^[2]^ These have opened up new treatment avenues for various malignancies; however, patients with t-NEPC and *BRCA* mutations are rare and the standard treatment is not established. In this report, we describe a unique case of t-NEPC with a *BRCA2* mutation, highlighting the sustained effectiveness of olaparib treatment. Additionally, we conducted a review of literature on similar cases of t-NEPC with *BRCA2* mutation treated with olaparib. This review aims to clarify the clinical features associated with *BRCA2* mutations in t-NEPC and to explore potential treatment strategies.

### 1.1. Case report

**Figure 1.**
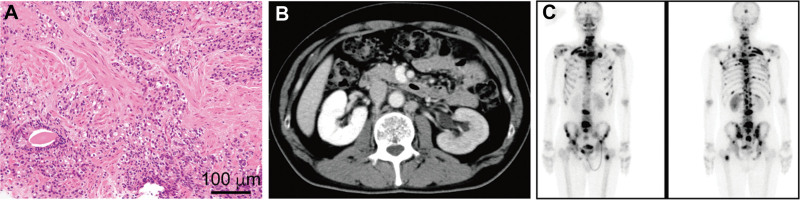
(A) Pathological findings of the prostate biopsy. Hematoxylin and eosin staining of the biopsy specimen revealed prostate adenocarcinoma (Gleason score 4 + 5 = 9). (B) Contrast-enhanced computed tomographic image showing enlarged para-aortic lymph nodes. (C) Bone scan revealing multiple bone metastases.

**Figure 2.**
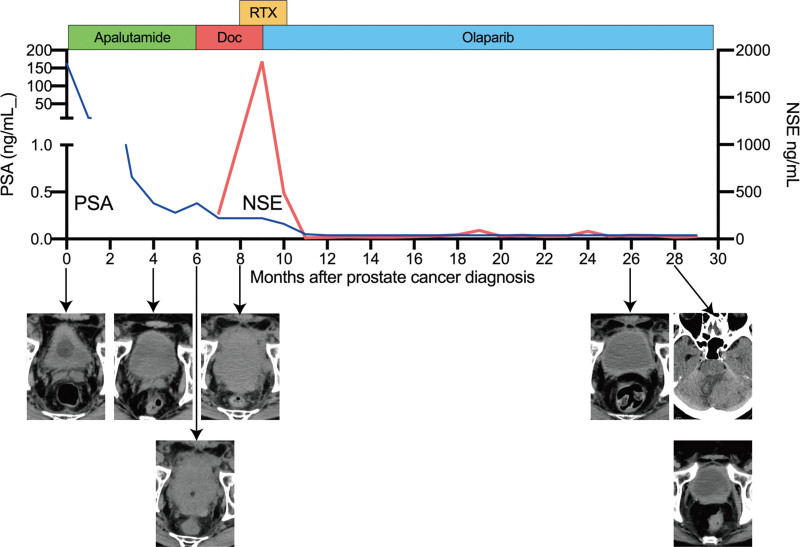
Clinical course following the diagnosis of prostate cancer. Representative images from each period are presented. Doc = docetaxel, NSE = neuron-specific enolase, PSA = prostate-specific antigen, RTX = radiotherapy.

**Figure 3.**
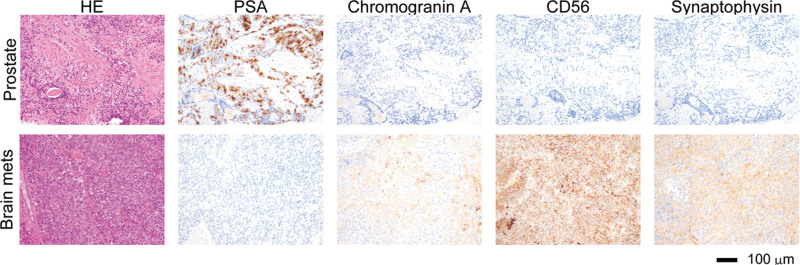
Pathological characteristics of prostate biopsy specimens and metastatic brain specimens. PSA and neuroendocrine tumor markers corresponding to the same locations as the HE staining are presented. HE = hematoxylin and eosin, PSA = prostate-specific antigen.

A 49-year-old male was presented to our hospital with a 6-month history of frequent urination. He had a family history of prostate cancer; his father had suffered from this condition. A rectal examination indicated a stony hardness. Blood tests revealed elevated alkaline phosphatase (489 U/L) and prostate-specific antigen (PSA) levels (162.3 ng/mL). Following a prostate biopsy, the patient was diagnosed with adenocarcinoma, with a Gleason score of 4 + 5 = 9 (Fig. 1A). Further imaging through computed tomography revealed multiple lymph node metastases and bone scintigraphy identified multiple bone metastases (Fig. 1B, C). Based on these findings, the patient was diagnosed with high-risk metastatic prostate cancer. Treatment with antiandrogen deprivation therapy and apalutamide was promptly commenced. However, 6 months later, the patient developed postrenal failure due to local progression and enlarged pelvic lymph node metastasis, yet without PSA progression (Fig. 2). Subsequently, after bilateral nephrostomy, docetaxel chemotherapy was administered, which resulted in an elevated level of neuron-specific enolase (NSE) (Fig. 2). Therefore, we added pelvic radiotherapy and submitted initial prostate biopsy specimens for cancer genome profiling. The cancer genome profiling revealed deletion of *BRCA 2* and *PTEN*, AR amplification, and the presence of the *TMPRSS2-ERG* fusion gene. After switching the treatment from docetaxel to olaparib, NSE levels decreased, and the patient achieved a complete response (CR) (Fig. 2). However, 18 months following the olaparib administration, brain metastases appeared despite the absence of pelvic tumor relapse, and the patient’s PSA levels remained low (Fig. 2). Subsequently, resection of the brain metastases using a gamma knife and whole-brain radiotherapy were performed. Pathological findings revealed metastatic cancer with positive neuroendocrine tumor markers and negative PSA, which differed significantly from the prostate biopsy specimen (Fig. 3). The patient died approximately 3 months after radiation therapy.

## 2. Discussion and literature review

Herein, we present a rare case of t-NEPC with a *BRCA2* mutation that showed the long-term efficacy of olaparib. There are 2 primary forms of drug resistance in cancer treatment: primary resistance and acquired resistance.^[3]^ Primary resistance refers to the inherent resistance present at the onset of treatment. This arises when the treatment fails to effectively target the factors causing cancer, leading to an inability to suppress signals that promote cancer growth. Conversely, acquired resistance develops subsequent to initial treatment, either due to the expansion of preexisting resistant clones under therapeutic pressure or through the adaptation of cells that develop resistance mechanisms during treatment. Acquired resistance emerges over time and is a consequence of alterations in cancer cells posttreatment. In this context, AR amplification and *BRCA2* mutations exemplify primary resistance, while t-NEPC is indicative of acquired resistance.^[3]^ Recent discoveries regarding prostate cancer have uncovered its capacity to transform into NEPC as a result of therapy, which is referred to as lineage plasticity.^[4,5]^ This case is significant because, although AR amplification in primary prostate cancer tissues is usually linked to resistance to ARSI.^[6]^ ARSI proved effective in treating tumors that produced PSA in this instance. Nevertheless, the patient underwent a recurrence in a relatively brief span, exhibiting a PSA-negative and NSE-positive profile. Metastatic cancer sites are known to possess a variety of genetic mutations, each exhibiting heterogeneity.^[7,8]^ This heterogeneity may be related to the reason why, despite the initial effectiveness of ARSI, the patient experienced a relatively quick recurrence and presented a different tumor marker profile.

In a previous case report,^[9]^ chemotherapy was a viable treatment option for NEPC. However, in this particular case, we highlight the long-term effectiveness of PARP inhibitors. Our search of the PubMed database using the keywords “neuroendocrine prostate cancer,” “*BRCA2* mutation,” and “Case” yielded 13 case reports.^[10–22]^ Of these, 5 cases from 4 articles involved individuals who had no prior history of cancer and were administered olaparib (Table 1).^[12,15,21,22]^ In terms of age and initial PSA levels, the patient was younger and had a significantly higher PSA value than did the other cases presented in the Table 1. Furthermore, specific mutations such as *PTEN*, AR amplification, and *TMPRSS2-ERG* fusion were observed in prostate cancer specimens. Unlike other cases in which all patients underwent platinum-based chemotherapy, this case did not involve such treatment but instead utilized radiation therapy. Notably, the longest duration of CR was achieved with olaparib. Additionally, the recurrence of brain metastasis in this case is a distinctive feature compared with the other cases presented.

**Table 1:** List of cases used in the literature review for neuroendocrine prostate cancer with BRCA2 mutation and olaparib treatment.

Case	Okubo et al^[22]^	Miyazawa et al^[21]^	Miyazawa et al^[21]^	Pandya et al^[15]^	Turina et al^[12]^	Current case
Case	1	2	3	4	5	6
Age	66	70	78	65	75	49
Initial PSA	17.6	40.8	14.2	95	9.23	162.3
Stage	cT4N0M1b	cT3bN1M1b	cT3bN0M0	cTxN1Mb	cT3bN0M0	cT3aN1M1b
Initial pathology	Small cell prostate cancer and adenocarcinoma	Adenocarcinoma	Adenocarcinoma	Adenocarcinoma	Adenocarcinoma	Adenocarcinoma
Other mutation	KEAP1, TP53	NA	NA	NA	ATM, Rb1, mTOR	*PTEN*, AR amp, *TMPRSS2-ERG* fusion
Treatment	CAB, Docetaxel, Carboplatin plus etoposide, Olaparib, Cabazitaxel	CAB, Carboplatin plus etoposide, Olaparib	CAB, Enzalutamide, Docetaxel, Cabazitaxel, Carboplatin plus etoposide, Olaparib	Abiraterone, Carboplatin plus etoposide, Olaparib	ADT, Enzalutamide, Carboplatin plus etoposide, Olaparib	Apalutamide, Docetaxel, Radiation, Olaparib
Duration of Olaparib	1 month	3 months	3 months	6 months	9 months	21 months
Best response by Olaparib	45.2% decrease in NSE level without any radiographic progression (SD)	Lymph node metastasis had shrunk (PR)	Lymph node metastasis had shrunk (PR)	Recurrent hepatic metastases (PD)	CR	CR
Follow-up period	2 years	4 years	4 years	5 years	3 years	2 years
Outcome	Death	Alive	Alive	Death	Alive	Death
Average age	67.1					
Average initial PSA	56.5					

Search for “neuroendocrine prostate cancer,” “*BRCA2* mutation,” and “case report”

“Only cases with ‘olaparib treatment’ and ‘without previous cancer history’ were selected.”

ADT = androgen deprivation therapy, CAB = combined androgen blockade, CR = complete response, PD = progressive disease, PR = partial response, PSA = prostate-specific antigen, SD = stable disease

The distinct features observed in the present case may be linked to the *BRCA2* mutations and radiation therapy. Recent studies on patient-derived xenografts of neuroendocrine prostate cancer with *BRCA* mutations have reported substantial tumor-suppressive effects by PARP inhibitors.^[23]^ This suggests a possible connection between *BRCA* mutations and t-NEPC as driver mutations. In addition, prior research has also indicated that olaparib may exhibit radiosensitizing effects, particularly for lung cancer.^[24]^

Consistent with the previous case report,^[9]^ we noted the presence of multiple metastatic lesions in the brain without local recurrences. Our findings underscore the challenges associated with achieving sufficient drug penetration across this barrier. Based on the previous report^[9]^ and our patient, recurrence of brain metastasis is a common occurrence with t-NEPC, underscoring the need for regular brain imaging examinations and not solely abdominal imaging.

## 3. Conclusion

The incidence of t-NEPC is on the rise, yet there is no established treatment protocol. Nonetheless, there is some indication that PARP inhibitors may be beneficial in cases where *BRCA* anomalies are present. We suggest that patients receiving olaparib for CRPC with *BRCA* mutations undergo regular brain imaging.

## Acknowledgments

We would like to thank Editage (www.editage.com) for the English language editing.

## Author contributions

**Conceptualization:** Rio Uehara, Daisuke Obinata.

**Writing—original draft:** Rio Uehara, Daisuke Obinata.

**Data curation:** Sho Hashimoto, Ken Nakahara, Hideaki Uchida, Toshiyuki Ishige.

**Investigation:** Tsuyoshi Yoshizawa, Junichi Mochida, Kenya Yamaguchi, Katsuhiro Miura.

**Writing—review and editing:** Masakuni Sakaguchi, Yoshinari Ozawa, Fumi Mori.

**Supervision:** Shinobu Masuda, Tomohiro Nakayama, Satoru Takahashi.
